# Inactivation of Enveloped Bovine Viral Diarrhea Virus and Non-Enveloped Porcine Parvovirus Using Low-Pressure Non-Thermal Plasma

**DOI:** 10.3390/life11121292

**Published:** 2021-11-24

**Authors:** Florian Le Bras, Gaëlle Carré, Yasmina Aguemon, Marius Colin, Marie-Paule Gellé

**Affiliations:** 1 Laboratoire “Biomatériaux et Inflammation en Site Osseux” (BIOS)—EA 4691, Université de Reims Champagne-Ardenne, SFR CAP-Santé, 51 Rue Cognacq-Jay, CEDEX, 51097 Reims, France; carregaelle1@gmail.com (G.C.); marius.colin@univ-reims.fr (M.C.); marie-paule.gelle@univ-reims.fr (M.-P.G.); 2 Texcell Company, 1 Rue Pierre Fontaine, CEDEX, 91058 Evry, France; yaguemon@texcell.fr; 3 UFR Odontologie, Université de Reims Champagne-Ardenne, 2 Rue du Général Koening, 51100 Reims, France

**Keywords:** non-thermal plasma, low pressure, virus inactivation, porcine parvovirus, Bovine viral diarrhea virus, oxygen plasma, oxygen-argon plasma

## Abstract

As the worldwide population has been experiencing since 2020, viruses represent a serious threat to global well-being. To avoid viral transmission through surgery or medical examination, sterilization of medical material is needed. From emerging sterilization processes, the use of non-thermal plasma (NTP) arises as a promising technique to efficiently reduce microbial burden on medical devices, including new complex polymers as thermosensitive ones. Thus, we evaluated the antiviral efficacy of a low-pressure NTP process taking place in a sealed bag. For this purpose, two different plasmas, O_2_ 100% plasma and Ar 80%–O_2_ 20% plasma, were tested against two viruses: the bovine viral diarrhea virus and the porcine parvovirus, surrogates of human hepatitis C virus and human parvovirus B19, respectively. The efficacy of both NTP treatments on viral load can be detected after only five minutes. Moreover, the longer the NTP treatments last, the more the load decreases. The most effective load reduction was obtained with a 120-min O_2_ plasma treatment inducing a minimum of four-log viral load reduction. So, this process demonstrated strong virucidal capacity inside a sealed bag and represents a very interesting opportunity in the field of fragile medical devices sterilization or disinfection.

## 1. Introduction

As the COVID-19 pandemic demonstrated, viruses represent a huge threat to humanity. Therefore, the control of the microbiological risk is of utmost importance. Transmission of viral particles mostly occurs through direct human contact with someone already colonized or infected by a virus. Depending on the virus’ nature, this transmission can operate through direct skin contact [1,2,3] or through aerosol droplets generated while coughing in the case of respiratory viruses such as influenza viruses or coronaviruses [4,5,6]. However, viruses can also spread through less obvious pathways such as an inert environment [7,8,9,10,11]. Indeed, contamination of surfaces by pathogens is systematic in the environment of infected people. On these surfaces, viruses can persist from hours to years [12,13], which implies the risk of cross-transmission through contact with them. Moreover, these contaminations are even more concerning when they occur on medical devices.

Thus, ensuring an efficient sterilization of medical devices is capital to guarantee patients’ safety. Several methods are currently used, the most frequent being high-pressure steam as it is cheaper and easier to perform. Nevertheless, this method is not adapted to many materials such as thermosensitive polymers or oxidable materials in electronic-related devices. Moreover, sterilization may also be performed through chemical treatment (ethylene oxide) or through ionizing radiation (gamma rays) [14]. Both techniques still present important limitations in spite of overcoming the temperature issue with high-pressure steam. Ethylene oxide requires long processing and ventilation times, and can potentially be carcinogenic for humans [15,16], while radiation treatments affect the properties of several types of polymers [17,18], require specific installations and are quite expensive. Thus, alternative disinfection and sterilization strategies are needed.

Known as the fourth state of matter, plasma and more particularly non-thermal plasma (NTP) arises as one of the most promising innovative sterilization methods. NTPs generated at atmospheric or low pressure are characterized by various active agents such as ultraviolet (UV) photons, negative and positive ions, free radicals as reactive oxygen species (ROS), reactive nitrogen species (RNS) or both (RONS) and excited atoms and molecules [19]. However, the nature of these agents varies substantially depending on the plasma techniques [20]. Indeed, to create plasma, there are so many processes using different electrical discharge methods such as atmospheric pressure plasma jet (APPJ), dielectric barrier discharge (DBD) [21]. These NTPs differ in terms of working pressure, frequency of the power source for plasma discharge, as well as gas nature and flow rate. Great interest has grown for NTP that do not exceed 60 °C and have shown their efficiency to inactivate various micro-organisms such as bacteria, spores, yeasts or viruses [21,22,23]. Still, a major obstacle persists: those techniques cannot keep un-conditioned items sterile after the end of the plasma treatment. This is a capital step to ensure items’ sterile state during transportation and storage before their use.

In previous studies [24,25,26], we investigated the efficacy of low-pressure O_2_, N_2_ and Ar NTPs. This technique allowed plasma activation with low energy and low gas flow (100 W and one standard cubic centimeter per minute (sccm)) compared to atmospheric conditions. Plasma was applied on preconditioned items that were contaminated with Gram-positive bacteria (*Staphylococcus aureus*), Gram-negative bacteria (*Pseudomonas aeruginosa*) and spores (*Bacillus subtilis*). The results highlighted the possibility to inactivate these strains regarding disinfection or sterilization norms of medical devices (AFNOR SPEC T71-902, NF EN ISO 15883, NF EN ISO 14937).

Then, the aim of this study was to evaluate whether this low-pressure NTP process could inactivate two surrogates of human viruses: the bovine viral diarrhea virus (BVDV), an enveloped virus surrogate of human hepatitis C virus, and the porcine parvovirus (PPV), a non-enveloped virus surrogate of human parvovirus B19.

## 2. Materials and Methods

### 2.1. Viruses and Cell Lines

Two viral strains have been used: Bovine viral diarrhea virus (BVDV, strain ATCC VR-534), as a single-stranded RNA enveloped virus and Porcine parvovirus (PPV, strain ATCC VR-742) as a single-stranded DNA naked virus. For the titration assay, BVDV was propagated in MDBK cells (ATCC CCL-22) and PPV in ST cells (ATCC CRL-1746). MDBK cells were cultured in Dulbecco’s modified Eagle medium (DMEM) F12 supplemented with 5% fetal bovine serum (FBS), 2% glutamine, and 1% gentamicin. ST cells were grown in Eagle’s minimal essential medium (EMEM) supplemented with 10% FBS, 2% glutamine, 1% gentamicin, 1% non-essential amino acid, 1% sodium pyruvate and 0.5% lactalbumin hydrolysate. Before use, viruses were expanded in cell cultures (according to Texcell’s standard operating procedures) and frozen at −70 °C.

### 2.2. Spiking Procedure

20 µL of thaw 2.58 × 10^8^ TCID_50_/mL BVDV suspension or 1.32 × 10^8^ TCID_50_/mL PPV suspension were spread onto a glass slide and allowed to dry for at least 15 min under a Biological Safety Cabinet until the slide was completely dry. The slides were taped into a plastic Petri dish which was placed inside a sterilization bag sealed right after.

### 2.3. Plasma Process

The plasma prototype device (Aurora, Loos, France) and the process have been detailed by Ben Belgacem et al. [25]. Briefly, a sealed sterilization bag (Südpack Medica, Ochsenhausen, Germany), containing the slides, was placed inside a steel vacuum chamber (35 L) and connected to the gas system. When pressure reached 1.45 × 10^−4^ mbar inside the chamber, the gas (O_2_ 100%) or the gas mixture (Ar 80%–O_2_ 20%) was injected continuously inside the bag through a Tyvek^®®^ membrane at a constant 1 sccm flow rate. The excess gas was released through the second Tyvek^®®^ membrane into the vacuum chamber. When the working pressure has reached 1.80 × 10^−4^ mbar in the vacuum chamber, a 100 W-radiofrequency was applied to the plate located at the bottom of the vacuum chamber to generate NTP, and the application of a magnetic field allowed to concentrate charged particles. Controlling the pressure difference between the vacuum chamber and the bag, the plasma is kept confined inside the bag. The temperature inside the bag was checked by surface temperature indicating strips (Thermographique 1; ThermoFisher Scientific, Waltham, MA, USA). After a full-time treatment temperature did not exceed 40 °C. At the end of the treatment, the chamber was put back at atmospheric pressure, the bag was disconnected, placed under sterility conditions and opened to apply the recovery procedure on slides.

### 2.4. Treatment Conditions

Three conditions were tested on both viruses. The first one is a low-pressure exposure during a full-time treatment duration (120 min). This condition presented no gas injection, no RF discharge and no magnetic field activation. The two other conditions were O_2_ 100% plasma or Ar 80%–O_2_ 20% plasma applied for 5 min, 15 min, 60 min and 120 min. For each condition two runs with three technical repeats were performed. As control samples, slides were kept under cabinet during a full-time treatment duration until recovery procedure.

### 2.5. Recovery Procedure

After plasma treatments, each slide was placed in a Petri dish, and 10 mL of culture medium was added to allow viral resuspension. Medium and slide were transferred into 50 mL polypropylene tube before 10 min of mechanical transverse agitation. Then the tube was vortexed, and the slide was rinsed three times with culture medium using pipette. The persistent smear was removed from the slide using scrapper. Then, the medium containing viral particles was collected and frozen at ≤−70 °C for future titration.

### 2.6. Titration Assay

The viral titer was determined by end-point titration assay. A first 96-well plate named Sample Dilution Plate was inoculated with serial 3-fold dilutions of defrosted BVDV or PPV eluates. Eleven dilutions were performed and eight replicates of each dilution were distributed per column. Next, another 96-well plate, a sample titration plate with adhered MDBK or ST cells in each well was inoculated well to well from the sample dilution plate and incubated to allow viral replication and infection of adjacent cells. Presence of infectious viral particles was then detected through crystal violet coloration of cells and observation of plaque forming unit (PFU).

The infectious titer was expressed as 50% tissue culture infective dose per milliliter (TCID_50_/mL). The viral inactivation of each condition was calculated as the difference of viral load between control (glass slides kept under cabinet) and the tested condition (low pressure, O_2_ 100% plasma or Ar 80%–O_2_ 20% plasma).

### 2.7. Statistical Analysis

To be relevant, statistical treatment of results had to be adapted according to the amount of viral infectious particles detected. Table 1 exposes the cases and subcases leading to the choice of the accurate statistical method.

Statistical analysis of the viral load reduction between O_2_ 100% plasma and Ar 80%–O_2_ 20% plasma at each treatment time has been performed with non-parametric Mann–Whitney. The results were considered as statistically significant when *p* < 0.05.

## 3. Results

The slides kept under cabinet for 120 min correspond to the control samples allowing the initial viral load determination.

### 3.1. PPV Inactivation by O_2_ and Ar/O_2_ NTP

**Figure 1:**
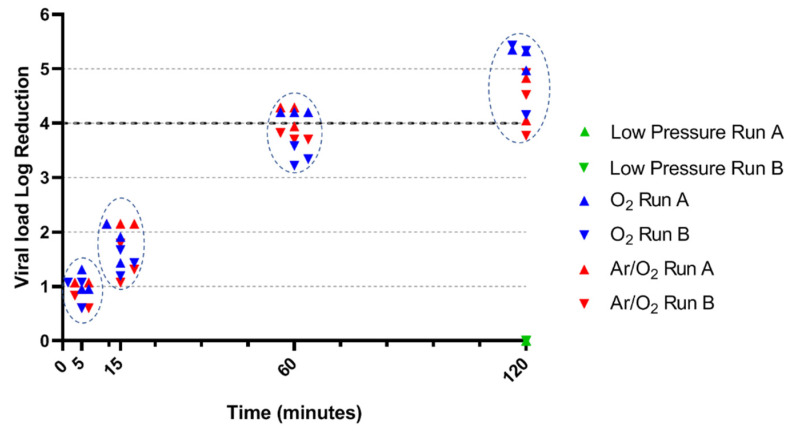
Antiviral efficacy of NTP against PPV. Circled dots underwent the same time of treatment, namely 5, 15, 60 or 120 min. Statistical comparison between O_2_ and Ar/O_2_ demonstrated no significant difference except for 120-min treatment.

As a control, the effect of low pressure was tested on both viral strains. A 120-min low-pressure treatment without plasma induced no observable reduction of the PPV load (0-log reduction) (Figure 1). On overall, O_2_ and Ar/O_2_ plasmas displayed similar results with a progressive reduction of the PPV load through the time of treatment. At 5 min, both plasma treatments already induced a PPV load reduction: O_2_ treatment led to a 0.60 to 1.31 log load reduction while Ar/O_2_ led to a 0.60 to 1.07 log load reduction (*p* = 0.3810). Both treatments performed similarly at 60 min (*p* = 0.3528), with a minimum of 3.82 log and 3.22 log PPV load reduction for Ar/O_2_ and O_2_ plasmas respectively. A trend appeared at 120 min (*p* = 0.0152): Ar/O_2_ plasma demonstrated a load reduction ranging from 3.77 log to 4.92 log, while O_2_ plasma systematically induced a load reduction higher than 4 log, ranging from 4.15 log to 5.43 log. Comparing gases antiviral effect for each treatment time, a significant difference was observed for 120 min treatment only (*p* = 0.0152). All other conditions showed no statistical differences between O_2_ and Ar/O_2_ treatments.

### 3.2. BVDV Inactivation by O_2_ and Ar/O_2_ NTP

**Figure 2:**
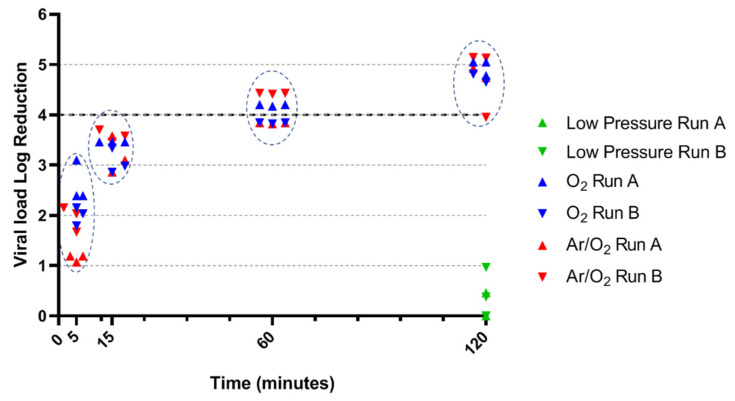
Antiviral efficacy of NTP against BVDV. Circled dots underwent the same time of treatment, namely 5, 15, 60 or 120 min. Statistical comparison between O_2_ and Ar/O_2_ demonstrated no significant difference except for 5-min treatment.

BVDV demonstrated slightly higher sensitivity than PPV when only exposed to low pressure. The viral load reduction was between 0 and 0.97 log after a 120-min exposure (Figure 2). The BVDV load quickly decreased through plasma treatment: after only 5 min, the load reduction ranged from 1.07 log to 2.15 log with Ar/O_2_ NTP, and from 1.79 log to 3.10 log with O_2_ NTP (*p* = 0.0216). The BVDV load continued to fall until 60 min, in similar ways for the two conditions. Thus, at 15 min, the load reduction was oscillating between 2.86 log and 3.46 log with O_2_ NTP and between 2.86 log and 3.70 log with Ar/O_2_ NTP (*p* = 0.2835). After 60 min, the load reduction was close to 4 log, with a minimum of 3.82 log load reduction for each condition (*p* = 0.5000). A 120-min treatment finally led to overall reductions close to 5 log. O_2_ NTP led to high efficacy, ranging from 4.65 log to 5.05 log, while one replicate exposed to Ar/O_2_ NTP demonstrated particularly low reduction of 3.95 log (*p* = 0.6623). Comparing gases antiviral effect for each treatment time, a significant difference was observed for 5-min treatment only (*p* < 0.05). All other conditions showed no statistical differences between O_2_ and Ar/O_2_ treatment.

## 4. Discussion

In the medical field, non-thermal plasma has been regarded as a promising sterilization process for two decades. Indeed, this technique could enable a high-level disinfection or sterilization while maintaining physical and mechanical properties of medical devices. In two previous studies [25,26], the antibacterial activities of NTP in low-pressure condition were investigated and demonstrated high efficiency against *S. aureus*, *P. aeruginosa* and *B. subtilis* spores. Here, we completed these studies by evaluating the antiviral activities of the same process.

Two different viruses were studied: BVDV, an enveloped virus with single-stranded RNA genome, as well as PPV, a non-enveloped virus with single-stranded DNA genome. After a 120-min low-pressure treatment (without plasma), the BVDV load was reduced from 0 to 1 log while no effect of this treatment was observed on PPV. In conclusion, low pressure cannot explain by itself the inactivation of these viruses. Moreover, the pressure is lower during a low-pressure exposure (10^−5^ mbar) than during a plasma treatment (10^−4^ mbar); so, the BVDV load reduction could be overstated.

Low-pressure O_2_ and Ar/O_2_ NTPs demonstrated high efficiency against the two viruses whatever the nature of the gas. Plasmas were particularly effective against BVDV in the early phases of the treatment (5 and 15 min), while the non-enveloped PPV demonstrated higher resistance at the same phases. After a 60-min NTP treatment, the residual load of both PPV and BVDV grew closer whatever the gas nature. A 120-min Ar/O_2_ NTP treatment did not systematically end up in a four-log viral load reduction of both viruses, unlike a 120-min O_2_ NTP treatment. The latter is thus seen as virucidal. Whereas the faculty of argon was said to potentiate another gas [27,28], the pure O_2_ plasma herein performed slightly better than the Ar/O_2_ plasma. This is consistent with the results we obtained during antibacterial tests where O_2_ led to better reductions than pure Ar [25].

The higher sensitivity of enveloped virus compared to non-enveloped virus could also be observed when they are exposed to different stresses. Thus, non-enveloped viruses such as Parvoviruses are well known to be resistant under environmental conditions [13] and under moist and dry heat treatments [29]. We observed a higher resistance of PPV compared to BVDV during the first phases of NTP treatments only. This difference could be explained taking into account the specific structures of both strains and their possible interactions with the different plasma species. Indeed, PPV is characterized by a protein capsid [30] whereas BVDV shows a phospholipid bilayer surrounding a protein capsid [31]. Although the mechanisms of atmospheric cold plasma on viruses are still unclear, the review by Filipić et al. [19] highlighted the key role of oxidative plasma species disrupting virus integrity. The membrane phospholipids of enveloped-virus are easily peroxidized during an oxidative stress initiated either directly by plasma RONS or by their interaction with UV radiations leading to the formation of ROS [32,33]. However, due to the presence of the protein capsid, it seems that UV radiations have difficulties in reaching and altering DNA. Despite this barrier, RONS could represent the essential mechanism of structural and genomic alterations of viruses because they affect lipids, capsid proteins, and nucleic acids [19,34]. Many studies have investigated the role of reactive species such as singlet O_2_ (^1^O_2_), ozone (O_3_), hydrogen peroxide (H_2_O_2_), ONOOH, ONOO^−^ and NOx to inactivate Feline calicivirus (FCV) and bacteriophages [34,35,36]. They showed that ^1^O_2_ had a key role to disrupt virus integrity by altering proteins of the capsid, and then damaged the nucleic acids. Regarding the inactivation of FCV, Yamashiro et al. [35] also highlighted the significant contribution of ONOO− as well as ONOOH, and the minor role of H_2_O_2_.

However, all the studies described in the review of Filipić et al. [19] concern atmospheric NTPs [19] while our process generated a NTP at 0.01 Pa. Some differences exist between atmospheric and low pressure NTPs as the generation of vacuum ultraviolet (VUV) in low pressure conditions [27,37]. These VUV are highly energetic due to their short wavelength (from 100 to 200 nm) and could potentially degrade viral genome. Thus, VUV and RONS may act together according to previously described mechanisms and may have a considerable impact on the viral inactivation. Further studies investigating on low-pressure NTP mechanisms will be crucial to determine what phenomenon among these represents the essential source of antiviral effect and what is the essential action site on the viral particle.

## 5. Conclusions

In conclusion, low-pressure O_2_ NTP is a promising method to inactivate enveloped and non-enveloped viruses. This process has the advantage of being able to treat preconditioned devices and is of great interest for thermosensitive ones. However, more physical studies are needed to better understand the antiviral mechanisms of low-pressure NTP. In addition, the effectiveness of our method against a particularly resistant virus, Porcine parvovirus, raises high expectations regarding the use of low pressure NTP against SARS-CoV-2. Indeed, SARS-CoV-2 is part of the coronavirus family which are known to be less resistant than porcine parvovirus. Thus, it would be interesting to evaluate the effectiveness of our process on SARS-CoV-2.

## 6. Patents

Popot, J.-M., and Gelle, M.-P. (2012). Device for Cold Plasma Sterilization of an Object, Such as a Medical Device, Particularly an Implant, and Method Using This Device. Available at: https://patentscope.wipo.int/search/en/detail.jsf?docId=WO2012038669 (accessed on 14 September 2021).

## Figures and Tables

**Table 1:** Choice of statistical treatment to determine viral titer according to the percentage of positive wells.

Case	Subcase	Titer (T)
Infectious particles detected ≥12.5% positive wells/total tested wells	N/A	T = T_SK_
Few infectious particles detected <12.5% positive wells/total tested wells	T_MaxL_ > LOD	T = T_MaxL_
	LOD > T_MaxL_	T = LOD
No infectious particles detected 0% positive well/total tested wells	N/A	T < LOD

T = titer retained for the calculation of the reduction factor; T_SK_ = infectious titer using the simplified Spearman–Kärber formula; T_MaxL_ = infectious titer using the maximum likelihood estimation; LOD = limit of detection using the Poisson formula with 95% precision; N/A = not applicable.
